# Antioxidant, Anti-Obesity, Nutritional and Other Beneficial Effects of Different Chili Pepper: A Review

**DOI:** 10.3390/molecules27030898

**Published:** 2022-01-28

**Authors:** Azrina Azlan, Sharmin Sultana, Chan Suk Huei, Muhammad Rizal Razman

**Affiliations:** 1 Department of Nutrition, Faculty of Medicine & Health Sciences, Universiti Putra Malaysia (UPM), Serdang 43400, Selangor, Malaysia; adachan91@gmail.com; 2 Research Centre of Excellence for Nutrition and Non-Communicable Diseases, Faculty of Medicine and Health Sciences, Universiti Putra Malaysia (UPM), Serdang 43400, Selangor, Malaysia; 3 Halal Products Research Institute, Universiti Putra Malaysia (UPM), Serdang 43400, Selangor, Malaysia; 4 Grain Quality Testing Laboratory, International Rice Research Institute (IRRI), Dhaka 1213, Bangladesh; m.s.sultana@irri.org; 5 Research Centre for Sustainability Science and Governance (SGK), Institute for Environment and Development (LESTARI), Universiti Kebangsaan Malaysia (UKM), Bangi 43600, Selangor, Malaysia

**Keywords:** chili pepper, anti-obesity, capsaicin, antioxidant properties, diet, beneficial effects

## Abstract

Fruits and vegetables are important components of a healthy diet. They are rich sources of vitamins and minerals, dietary fibre and a host of beneficial non-nutrient substances including plant sterols, flavonoids and other antioxidants. It has been reported that reduced intake of fruits and vegetables may increase the risk of non-communicable diseases (NCDs). Chili pepper, is a common and important spice used to enhance taste and nutrition. Over the years, reports have shown its potential as antioxidant and an anti-obesity agent. Obesity is a serious health concern as it may initiate other common chronic diseases. Due to the side effects of synthetic antioxidants and anti-obesity drugs, scientists are now focusing on natural products which produce similar effects to synthetic chemicals. This up-to-date review addresses this research gap and presents, in an accessible format, the nutritional, antioxidant and anti-obesity properties of different chili peppers. This review article serves as a reference guide for use of chili peppers as anti-obesity agents.

## 1. Introduction

Nowadays, obesity is a worldwide health concern. Obesity is a risk factor for heart disease, stroke, diabetes mellitus, osteoarthritis (OA), and hypertension [1]. There are various options to cure obesity such as weight management, medication, and surgery. Some diet therapy such as keto, low-carb, high-protein diet, high-fat diet, and low energy diets are used for obesity treatment, using established behaviour-change techniques and social support on a short-term basis [2]. These practices do not harm a person’s health if maintained on a long-term basis. Compared to a non-ketogenic diet, a ketogenic diet has less sustainability [3]. 

Weight management in the form of lifestyle changes does not generally produce marked or sustainable weight loss [4]. In contrast, surgery is only suggested for patients with severe obesity, especially when the implementation of dietary changes and physical activities does not produce significant weight loss [5]. For example, weight loss surgery, such as bariatric surgery, is effective in term of weight loss, but can cause surgical complications and frequent reoperation [6]. 

Vegetables are an indispensible part of a healthy diet. A healthy diet high in fruits and vegetables and low in fat, sugars and salt may help to reduce the risk of cardiovascular diseases and excess weight gain [7]. The chili pepper, a plant that belongs to the capsicum genus, is an important spice that is widely used as a vegetable plant. Chili pepper is also used in a therapeutic diet due to the activities of unique components, capsaicin and capsaicinoids, that contribute to the pungent scent of hot chili pepper. Recent studies on chili peppers have focused on their beneficial functions as potential anti-tumor, anti-cancer, antioxidant, anti-obesity agents [8,9,10]. 

It has been documented that capsaicin can act as a potential agent for anti-obesity caused by adipogenesis of cells and oxidative stress [9]. However, there is little evidence concerning the effect of capsaicin’s anti-obesity properties. Additionally, the antioxidant content and activities of different chili pepper varieties have not been documented in detail. This review is an attempt to redefine the importance of these compounds as possible solutions to the problem of obesity and to explore their potential for the benefit of human health. 

## 2. What Are Chilis?

Chilis belong to the genus *Capsicum*, the most important genus in the family of Solanaceae (Table 1). Chili fruits are botanically known as berries. Unlike most berries, chili seeds are not embedded in the fleshy pericarp but are located in the placenta. Chili fruits vary in shape in different species. Different chili species have their own unique shapes [11,12]. The extreme variability in fruit characters results in difficult classification of chili crops. For commercial purposes, the classification of chili products is usually based on variations in pungency, color, flavor, their uses, and the size and shape of the fruits [12]. *Capsicum annuum* is the most widely cultivated and economically important *Capsicum* species. *Capsicum annum* includes cultivars such as sweet peppers, Jalapeno, pablano, cayenne, ancho, and Serrano types [12]. *Capsicum annuum* is a species of the plant genus which native to southern North America and northern South America. It is a small herb that can grow up to 1 m tall. Leaves are mostly ovate, ovate-lanceolate, or oblong-ovate. Chili flowers are small in size, white or purple-tinged in color. Most fruits are green, but some can be red or orange and can grow up to 15 cm, while seeds are mostly pale-yellow in color, reniform or discoid, and 3–5 mm in size. Most chili plant grows in tropical climates, as they need a warm, humid climate to survive [12].

The pedicels are normally pendant, but sometimes erect with the fruit kept upright until maturity. A good example of upright *C. annuum* is the *cili kulai* cultivar in Malaysia. The color of fruit changes from unripe dark green, green, yellowish or purplish, to the ripening color of red or orange. *C. annuum* has a variety of pungencies, ranging from 0% capsaicin content (bell pepper) to 0.9% (red chili cultivars). *C. frutescens* is a short-lived perennial herb. Depending on climate and growing conditions, the height of *Capsicum frutescens* is between 1–4 feet. The leaves measure 2½ inches long and 1 inch wide. The leaves are elliptical, slightly leathery and dark green in color. The flowers are typically white in color with a conical or funnel form and having five petals. The fruits are normally 10–20 mm long and 3–7 mm in diameter [13]. The fruits are green when immature and purple, red, or orange when ripe [14]. Cultivars of *frutescens* are common in Malaysia due to the long ripening period and the presence of volatile compounds in the chili flesh. Finally, *C Chinense Jacq.* includes some of the most pungent peppers, commonly recognized as habanero and Scotch bonnet types. The species is commonly cultivated in the Amazon region and is widespread in tropical America. *C Chinense Jacq.* has a very different fruit color (orange, red, and brown) and shape, resembling an isometric lantern with a squashed shape. There are also elongated and pointed cultivars. The fruit flesh is firm. The seeds are straw-colored and the same size as those of *C. frutescens*, but are bigger than *C. annuum* seeds [12].

### Uses of Chilis

Chili peppers were probably first used as medicinal plants before use for cooking. For example, the Mayans used chilis to treat asthma, coughs, and sore throats [15]. In Columbia, the Tukano group uses chili peppers to relieve a hangover [16]. The Aztecs and Mayans mixed pepper with maize flour to produce chillatolli, commonly used as a therapeutic diet to cure the common cold. The Teenek (Huastec) Indians of Mexico used chili peppers to treat infected wounds. Some other uses include putting red crushed fruits on the feet to cure athlete’s foot fungus, and to cure snakebites by making a drink from boiled green fruit [17]. Medicinally, capsaicin is used to alleviate pain [18]. At present, it is widely used as a medication for arthritis. In addition, capsaicin-containing cream is used to reduce post-operative pain in mastectomy patients [18].

## 3. Nutritional Value of Chilis

### 3.1. Vitamin Content of Chilis

Chili peppers are known to be one of the major sources of carotenoids, precursors of vitamin A and ascorbic acid (vitamin C). They contain high levels of carotenoids and L-ascorbic acid at maturity, having 0.33–336 RE/100 g RDA for provitamin A and 124–338% of the vitamin C activity [12]. Among various vitamins, ascorbic has strong antioxidant properties towards free radicals because of its reducing power due to its enediol structure, which is conjugated with the carbonyl group in a lactone ring [19]. Thus, it is often involved in the study of degenerative or chronic diseases prevention, as free radicals are major factors in these diseases. The concentration of vitamin C is dependent on fruit maturity and pungency. Ripe chili pepper fruit with high pungency levels possesses a high content of vitamin C [20].

Some studies also show that vitamin C is a potential component in managing body weight. In a screening study for phytochemical and antioxidants among Egyptian chili varieties [21], it was found that fresh chilis are a rich source of vitamin C. The increased level of vitamin C is associated with an increased stage of maturation. As reported by Bertao et al. [22], during a 3-year follow-up on 1983 adults aged 19–70, the participants with the highest total antioxidant capacity score had a lower weight and abdominal gain. Besides, there was an improvement in the cell viability of adipocytes from white adipose tissues of lean and obese rats after vitamin C treatments [23].

### 3.2. Bioactive Compounds in Chilis

Chili fruits vary in size, shape, color, flavor, and pungency. This variation depends on the species, cultivar, growing conditions, fruit maturity, and post-harvest handling [24]. The level of pungency depends on the concentration of capsaicinoids, mainly capsaicin. On average, chili pepper contains 30 to 600 ppm, with 600 to 13,000 ppm capsaicin. Capsaicinoid chemicals provide the distinctive tastes in *C. annuum* variants [25]. Moreover, capsaicin is responsible for the burning sensation, which in extreme cases can last for several hours after ingestion.

The antioxidative and antimicrobial properties of many plant extracts are of great interest, as there is a growing tendency to replace synthetic antioxidants with natural ones as natural additives used in both academic and industrial research [26]. The bioactive compounds in the species of *Capsicum* mainly consist of capsaicin, 6,7-dihydrocapsaicin, homodihy-drocapsain, nordihydro-capsaicin and homo-capsaicin [27]. Capsicum also is a good source of bioactive compounds, such as flavonoids, phenolic acids, carotenoids, and ascorbic acid. These compounds have been reported to have antioxidant and anti-inflammatory activities [28]. They are also important components for building up and maintaining the human immune system [29]. On the other hand, Capsaicin, chemically identified as 8-methyl-N-vanillyl-6-none, is the main compound in the genus, together with a group of similar substances called capsaicinoids. These compounds have been shown as potent antioxidant agents in the plant. Concerning their biofunctional activities, capsaicinoids exhibit both pharmacological and physiological actions. For instance, capsaicinoids act against high cholesterol levels and obesity, show anticancer effects, and are used to treat arthritis pain [30]. Capsaicin also possesses antimicrobial properties, which suggests its use as a potential natural inhibitor of pathogenic microorganisms in food [31].

Similarly, capsaicin is the major active component from the capsaicinoids. This is a component unique to the Capsicum genus [32]. The presence of capsaicin in chili is determined by a major gene, but it is the action of polygenes acting in a cumulative manner that determines the various degrees of pungency. Capsaicin can be absorbed well when administered topically or orally, reaching up to 94% absorption due to its unique chemical structure [33,34].

Capsaicin has many pharmacological benefits to humans, such as treating chronic pain syndrome, anticancer effects, a hypoglycemic effect, treatment of hypertension and ischemic heart disease, antimicrobial effects, and anti-obesity effects [35].

Recent progress has focused on the chemo-preventive effect of capsaicin, reflecting its anti-growth activity against various cancer cells, such as breast cancer, prostatic cancer, colorectal cancer, lung cancer, gastric cancer, and pancreatic cancer. Capsaicin exerts its cytotoxic action by activating an array of signaling mechanisms, including the generation of reactive oxygen species (ROS), up-regulation or activation of p53 [35], suppression of signal transducers and activating the transcription (STAT) family of proteins [36] and the NF-kB pathway [36]. The selectivity of the anti-cancer effects towards malignant cells is shown in [35].

### 3.3. Phytonutrients in Chilis

Carotenoids are lipophilic yellow-orange-red pigments that y gives color to photosynthetic plants. In Capsicum spp., carotenoids exist in various forms and structures at different maturity stages. For example, in green-colored chili pepper fruit, a combination of chlorophyll and carotenoid is found in the chloroplast. At the intermediate maturity stage, zeaxanthin, α- and β-carotene, β-cryptoxanthin, and lutein are the major compounds that contribute to the orange or yellow color. When the chili pepper ripens, the carotenoid pigments transform into 5,6-epoxide, capsorubin, capsanthin, and capsanthin, which produce the red pigment [36].

Animals are not able to synthesize carotenoids de novo but must obtain them by dietary intake. Carotenoids can provide body tissues protection against light and oxygen. As reviewed by Arimboor et al. [37], red chili pepper is considered one of the major sources of β-carotene. The red color of chili pepper is caused by carotenoids. The carotenoid composition in chili pepper ranges from 0.1 g to 3.2 g/100 g dry weight, and varies with different varieties, maturation, extraction method, and agro-climatic condition. In a previous study [38] it was reported that the red color in the chili pod is imparted by a group of unique keto carotenoids with κ-ring as end groups, including capsanthin, capsorubin, and cryptocapsin. Maoka et al. [39] reported that carotenoids bearing κ-ring as end groups have strong oxygen scavenging potential.

According to a study conducted in 2005, Collera et al. showed the major identified carotenoids in Mexican chilis varieties were β-cryptoxanthin and β-carotene. Both have provitamin A activity. Studies show that Vitamin A is a potential anti-obesity agent by reacting with adipocytes [40].

The concentrations of flavonoids, total soluble reducing equivalents, and phenolic acids increase with fruit maturity. According to Jeon [41], high levels of flavonoids with constant levels of ascorbic acid and caffeic acid provide enhanced antioxidant activity. Nagy et al. (2015) [42] reported that vanillic acid-derivatives, catechin, narigenin-diglucoside, and flavonoids are the dominant polyphenols in chili peppers. The only non-flavonoid phenolic acid detected is vanillic acid. At the same time, Maksimova [43] found that quercetin rhamsoside and quercetin glucosides are the most abundant polyphenolic compounds in C. annuum L. extracts.

In 2008, Sim and Sil [44] reported that red pepper pericarp and seed extracts showed strong antioxidant activity. The red pepper pericarp has a high total flavonoid content (TFC) and total phenolic content (TPC). Its strong antioxidant activity is due to strong ferrous chelating activity and free radical scavenging activity, whereas the red pepper seed has high scavenging strength against the superoxide anion radical, and high ABTS radical scavenging activity. Thus, red pepper seed extract is a potent antioxidant agent.

## 4. Antioxidant Activities of Chilis: An Overview

Chili peppers are reported to contain moderate to high levels of phytochemicals including neutral phenolics and flavonoids that are important antioxidant components of a plant-based diet, providing health benefits beyond basic nutrition. These compounds possess biochemical and pharmacological effects including anti-inflammation, anti-allergy, anti-oxidation activities, and may reduce the risk of degenerative diseases [45].

In addition, antioxidants are also associated with a reduced rate of heart disease mortality [46] and reduced incidents of pharynx, mouth, oesophagus, stomach, colon and lung cancers, as well as premature aging [47].

According to the findings of [48], capsaicin inhibits radiation-induced biochemical alterations, including protein oxidation and lipid peroxidation. This study suggests that capsaicin found in Chili pepper can act as a radio-protector and antioxidant in physiological systems.

In intervention studies of capsaicin microemulsions both pure capsaicin and capsaicin microemulsions showed higher inhibitory capacities than the synthetic antioxidant BHT. This formulation can be used as a natural preservative in meat product preparations [49,50]. Hossain et al. (2008) [48] showed that the antioxidant activities of capsaicin in chili are comparable to synthetic antioxidants such as butylated hydroxyanisole (BHA) and butylated hydroxytoluene (BHT).

In addition, the application of capsaicin consumption provides a synergistic effect with certain therapeutic drugs. For example, the combination of 5-fluorouracil (5-FU) and capsaicin enhances drug sensitivity of cholamgiocarcinoma (CCA), which is a type of cancer with multi-drug resistance, by suppressing the growth of the cancer cells [46,51].

### 4.1. General Concepts of Antioxidants

Recently, scientists have given a great deal of attention to the role of plant-derived antioxidants in human health. In general, the term antioxidant is defined as a molecule that can prevent or delay the oxidation of easily oxidizable materials (such as lipids or fats) in a significant way [10,52,53]. However, it has been documented that lipids are not the only materials that undergo oxidation, since DNA and proteins are also susceptible to oxidation processes [54,55]. Antioxidants can be defined as substances that in low quantities, when compared to those of oxidizable substrates, are able to delay or prevent the substrate from becoming oxidized [56]. The anti-cancer property of capsaicin was further justified by Oyagbemi et al. in 2010 [56]. This study showed that capsaicin blocks the translocation of nuclear factor kappa β (NF-kβ), activator protein-1 (AP-1), and the signal transducer and activator of the transcription (STAT 3) signaling pathway that are required for carcinogenesis. Capsaicin induces apoptosis and cell cycle arrest due to the generation of ROS.

In brief, an antioxidant can be considered as a substance capable of repairing systems such as iron-transporting proteins by inhibiting a specific oxidizing enzyme that may react with oxidizing agents. However, there is no accepted international definition for the term antioxidant, and there is no universal ‘best’ antioxidant. For instance, ascorbate is well known as a protector against plasma lipid peroxidation caused by tobacco smoke, but is not able to protect against plasma protein damage [11,57].

### 4.2. Potential Anti-Obesity Mechanisms and Compounds in Chilis

A successful weight-loss agent should increase energy expenditure or reduce energy intake without adverse side effects. As reviewed by Jeon [41] several strategies have been applied for the development of anti-obesity agents, including induction of adipocyte apoptosis, reducing adipogenesis, increasing lipid metabolism, increased energy expenditure and reduced energy intake.

Most energy expenditure is associated with basal metabolism rate (BMR), which remains constant during the daytime, except for heavy physical exercise. By contrast, energy intake is highly variable during the day. Therefore, an imbalance between energy intake and BMR occurs. This leads to the occurrence of obesity if there is a positive balance of energy intake to BMR. To control energy intake and body weight, a potent anti-obesity agent should delay the initiation of hunger, and reduce the amount of consumed food by affecting satiation signaling [1]. Adipocytes, also known as lipocytes or fat cells, are mostly composed of adipose tissue, and have an important role in controlling lipid metabolism by storing energy as fat, and releasing energy through lipolysis.

#### 4.2.1. Obesity

Obesity is a complex medical condition. Recently, the incidence of obesity has become a major public health concern with incalculable social costs, and is increasing at an alarming rate [1,57]. Obesity is caused by the interaction of dietary effects, a sedentary lifestyle, and environmental and genetic factors which favors a chronic positive energy balance leading to increased body fat mass.

Obesity is recognized as a worldwide health crisis because of its predominant effects on worldwide mortality, morbidity, and finance. In addition, obesity is a risk factor for some common chronic diseases such as diabetes mellitus, heart disease, osteoarthritis, hypertension, stroke, and others [58].

In Malaysia, there is an increasing rate of obesity. In 2014, a study published by the British medical journal The Lancet said that Malaysia was rated the highest (45.3%) in Asia for obesity, followed by South Korea (33.2%), Pakistan (30.7%) and China (28.3%) [1].

In 2013, the National Health and Morbidity Survey (NHMS) reported that 4.4 million Malaysians were obese. The obesity prevalence showed an increasing trend over time. According to the NHMS in 2015, there were an estimated 5.6 million adults aged 18 and above that were overweight and 3.3 million Malaysians were obese. Hence, it is estimated that obese Malaysians make up 17.7 percent of the Malaysian adult population, while those who are categorized as overweight make up 30 percent. If added together, almost half the adult populations of Malaysia are either overweight or obese. Over the years, the obesity prevalence has been highest in the age category of 50–59 years, accounting for 60.2% of the overall overweight and obesity population in Malaysia [59].

Besides the adult population, the age group below the age of 18 has also shown an increasing trend over the years. According to the reports of NHMS in 2011 and 2015, the obesity prevalence among children below 18 years old increased from 6.1% to 11.9% [59]. In the latest NHMS study in 2019, one in two adults in Malaysia were overweight or obese, the highest category being females [59].

#### 4.2.2. Anti-Obesity Activities of Chilis: An Overview

The potential of natural products for treating obesity is under exploration. Natural products be excellent alternative strategies for developing safe and more effective anti-obesity drugs [60]. A variety of natural products have been widely used in treating obesity including isolated pure natural compounds and crude extracts that can induce bodyweight reduction and prevent diet-induced obesity [1].

Some dietary phytochemicals have been documented as anti-obesity agents because they may stimulate lipolysis, suppress the growth of adipose tissue, induce apoptosis of existing adipocytes, and inhibit differentiation of preadipocytes, thereby reducing adipose tissue mass.

Adipocytes play a central role in the maintenance of energy balance and lipid homeostasis by releasing free fatty acids and storing triglycerides in response to changes in energy demands [27]. Natural products that specifically target adipogenesis inhibition have been considered to have promising potential in obesity treatment. For example, polyunsaturated fatty acids (PUFAs) act as signal transducing molecules in adipocyte differentiation [27]. PUFAs plays a central role in suppressing lipogenesis and regulating adipocyte differentiation through suppression of late-phase adipocyte differentiation [61,62] Numerous natural products have apoptotic effects on maturing pre-adipocytes, such as capsaicin, linoleic acids, resveratrol, genistein, conjugated, and quercetin [61].

Chronic low-grade inflammation (CLGI) is one of the factors responsible for the development of obesity. Systematic CLGI damages pancreatic beta-cells, disrupts insulin action, and induces glucose intolerance in obesity. As reported in a study conducted by Kang et al. [50], dietary capsaicin significantly lowered the number of liposaccharides (LPS)-producing families and reduced high-fat diet-induced CLGI associated with anti-obesity properties.

Capsaicin also significantly decreased the amount of glycerol-3-phosphate dehydrogenase (GDPH) activity and intracellular triglyceride in 3T3-L1 adipocytes, and inhibited the expression of PPARγ, C/EBPα, and leptin [52]. This mechanism is supported by Lee et al. [62], who showed that 0.075% capsaicin decreased lipid accumulation in mesenteric and epididymal adipose tissue in high-fat diet-induced obese mice. Besides, the application of capsaicin in food formula significantly lowered the serum level of glucose, cholesterol, and triglycerides in mice.

In a recent study, capsaicin in C. annuum contributed to thermogenesis, which can increase energy expenditure. When capsaicin application was tested on 3T3-L1 adipocytes, it decreased intracellular lipid content and was involved in thermogenesis [63]. As reported by Kang et al. [50] dietary capsaicin can reduce metabolic dysregulation in obese/diabetic KKAγ mice by enhancing the expression of adiponectin and its receptor. Capsaicin also exerts an anti-proliferative effect that prevents the 3T3-L1 preadipocytes differentiating into mature adipocytes. In the same study, it was reported that capsaicin significantly downregulated transcription factors, especially PPARγ. Hence, the capsaicin content in chili may contribute to the maintenance of body weight and prevent the development of obesity [52].

#### 4.2.3. Transient Receptor Potential Cation Channel Subfamily V Member 1 (TRPV1 Receptor)

TRPV1 is a heat-sensitive ion channel that is mostly involved in pain sensation. It plays an important role in the human protective system, which helps us to avoid pain and heat [63]. TRPV1 is a protein encoded by the TRPV1 gene in humans and is also known as the capsaicin receptor and the vanilloid receptor 1 [64]. It is commonly found in the nociceptive neuron of the peripheral nervous system. It is a non-selective cation channel for capsaicin, calcium (Ca^2+^), and sodium (Na^+^) ion movement into sensory cells during activation [65]. The transmembrane core region of TRPV1 contains six transmembranes per subunit (S1–S6), and exhibits various structural features, each of them binding specifically to different types of cations [66]. In other words, TRPV1 receptors have distinct activation pathways for the specific types of substrate.

TRPV1 receptors play an important role in sensing of pain and detectors for chronic inflammatory pain, brain inflammation, and neuropathic pain [63]. Therefore, to relieve pain, inhibition of TRPV1 expression has become the therapeutic target for many compounds or drugs [63]. In addition, an increased influx of Ca^2+^ and Na^+^ ions during the occurrence of epilepsy makes TRPV1 a therapeutic target for antiepileptic action [67].

However, in a recent study, the activation of the TRPV1 receptor in the human body was suggested as a novel strategy for treating diet-induced obesity by control of food intake, and enhancement of energy metabolism and expenditure [68]. In contrast, the removal of TRPV1 was reported to result in potent anti-obesity properties. Lee and co-workers [69] found that, at the same HFD, TRPV1 knockout mice became more obese than the wild-type mice. The TRPV1 knockout mice also developed insulin and leptin resistance compared to the control. Since TRPV1 has reactivity toward oxidative stress and caused inflammation, Marshall et al. [70] suggested that deletion of TRPV1 may protect against obesity-induced hypertension, and the TRPV1 channel may cause the development of cardiometabolic syndrome.

#### 4.2.4. Capsaicin and TRPV 1

Capsaicin is a TRPV1 agonist that triggers the activation of TRPV1 mechanisms. The activation of TRPV1 by capsaicin causes pain and a burning sensation in the body. At the same time, it also helps in body temperature regulation [71].

In 2007, an animal study conducted by Zhang et al. [72] found that oral administration of capsaicin for 120 days was able to prevent the development of obesity in male wild-type mice but not in TRPV1 knockout mice. This study showed that the activation of TRPV1 channels by capsaicin helped in adipogenesis and obesity prevention. Besides, activation of TRPV1 in neurons of the hypothalamus was found to decrease food intake, suggesting one of the effects of TRPV1 in weight reduction [73]. Leung et al. [74] reported that mice that consumed a capsaicin-containing diet had increased mRNA expression of TRPV1 receptors in adipose tissue, and a reduction of 24% of visceral fat. This finding is supported by Choowanthanapakorn et al. [75], and further proves the role of capsaicin-induced TRPV1 activation in metabolic regulations that may be effective in weight-reducing treatments.

Activation of TRPV1 receptors by capsaicin also triggered anti-cancer effects in breast cancer, inhibiting cancer cell growth and inducing apoptosis and necrosis in the target cells (Weber et al., 2016). In addition, capsaicin-induced TRPV1 activation is reported to have a positive synergistic effect with pirarubicin (THP), which is an anti-cancer drug used to treat bladder cancer [76,77].

## 5. Conclusions

Obesity is characterized by chronic low-grade metabolic inflammation and may be regulated by the control of preadipocytes differentiation. Antioxidants, including polyphenol, vitamin C, and flavanol molecules, are potent radical scavengers. Reactive oxygen species and free radicals are the main factors that initiate pathological conditions such as inflammation, metabolic disorder, and carcinogenesis.

Bioactive compounds and vitamin C present in chili pepper are of great therapeutic importance as they have anti-inflammatory activities on preadipocytes differentiation and cellular oxidation. Adipogenesis is the process of cell differentiation by which preadipocytes become adipocytes, and is caused by the reduction of mRNA, protein, the activity level of peroxisome proliferator-activated receptor (PPARγ) and enhancer-binding protein (C/EBP). Capsaicin molecules can trigger the activation of TRPV1 mechanisms. They are the key transcription factors that regulate adipocyte differentiation and lipid synthesis. Thus, a potential anti-obesity agent can reduce the rate of adipogenesis to a very low level.

## Figures and Tables

**Table 1:** Taxonomy of Chilis.

Kingdom	Plantae
Sub-kingdom	Tracheobionta (vascular plant)
Superdivision	Spermatophyta (seed plant)
Division	Magnoliophyta (flowering plant)
Class	Magnoliopsida (dicotyledons)
Subclass	Asteridae
Order	Solanales
Family	Solanaceae (potato family)
Genus	*Capsicum L.* (pepper)
Species	*C. annuum*, *C. baccatum*, *C. chinense*, *C. pubescens*, *C. frestescens*

## Data Availability

Not applicable.
